# Inotuzumab ozogamicin for relapsed/refractory acute lymphoblastic leukemia: outcomes by disease burden

**DOI:** 10.1038/s41408-020-00345-8

**Published:** 2020-08-07

**Authors:** Daniel J. DeAngelo, Anjali S. Advani, David I. Marks, Matthias Stelljes, Michaela Liedtke, Wendy Stock, Nicola Gökbuget, Elias Jabbour, Akil Merchant, Tao Wang, Erik Vandendries, Alexander Neuhof, Hagop Kantarjian, Susan O’Brien

**Affiliations:** 1 Dana-Farber Cancer Institute, Boston, MA 02215 USA; 2 Cleveland Clinic Taussig Cancer Institute, Cleveland, OH 44106 USA; 3 University Hospitals Bristol, Bristol, BS1 3NU UK; 4 Universitätsklinikum Münster, 48149 Münster, Germany; 5 Stanford Cancer Institute, Stanford, CA 94304 USA; 6 University of Chicago, Chicago, IL 60637 USA; 7 Goethe University Hospital, 60596 Frankfurt, Germany; 8 University of Texas MD Anderson Cancer Center, Houston, TX 77030 USA; 9 Cedars Sinai Medical Center, Los Angeles, CA 90048 USA; 10 Pfizer Inc, Cambridge, MA 02139 USA; 11 Pfizer Pharma GmbH, 10785 Berlin, Germany; 12 Chao Family Comprehensive Cancer Center, University of California, Irvine, Orange, CA 92697 USA

**Keywords:** Acute lymphocytic leukaemia, Acute lymphocytic leukaemia, Targeted therapies

## Abstract

Adults with relapsed/refractory acute lymphoblastic leukemia (R/R ALL) have a poor prognosis, especially if disease burden is high. This post hoc analysis of the phase 3 INO-VATE trial examined the efficacy and safety of inotuzumab ozogamicin (InO) vs. standard of care chemotherapy (SC) among R/R ALL patients with low, moderate, or high disease burden, respectively, defined as bone marrow blasts (BMB) < 50% (*n* = 53 vs. 48), 50–90% (*n* = 79 vs. 83), and >90% (*n* = 30 vs. 30). Patients in the InO vs. SC arm with low, moderate, and high BMB%, respectively, had improved rates of complete remission/complete remission with incomplete hematologic recovery (74% vs. 46% [*p* = 0.0022], 75 vs. 27% [*p* < 0.0001], and 70 vs. 17% [*p* < 0.0001]), and improved overall survival (hazard ratio: 0.64 [*p* = 0.0260], 0.81 [*p* = 0.1109], and 0.60 [*p* = 0.0335]). Irrespective of BMB%, cytopenias were the most common treatment-emergent adverse events, and post-transplant veno-occlusive disease was more common with InO vs. SC. Patients with extramedullary disease or lymphoblastic lymphoma showed similar efficacy and safety outcomes. This favorable benefit-to-risk ratio of InO treatment irrespective of disease burden supports its use in challenging and high disease burden subpopulations. INO-VATE is registered at www.clinicaltrials.gov: #NCT01564784.

## Plain language summary

Acute lymphoblastic leukemia (ALL for short) is a type of blood cancer where the body makes too many immature white blood cells called lymphoblasts. This study involved people with ALL whose cancer had returned after, or stopped responding to, previous treatment. These people received either inotuzumab ozogamicin (InO for short) or standard chemotherapy. Researchers divided people into groups, based on the level of lymphoblast cells they had in their bone marrow (called disease burden): low, medium, or high disease burden. In this study, compared with people who received standard chemotherapy, people who received InO were more likely to have no signs of their cancer (called remission), live to the end of the study, and/or reach the end of the study without their cancer getting worse. The researchers saw these results across all disease burden groups. For people who received InO, those with high disease burden were equally as likely as those with low disease burden to achieve remission, and/or experience medical problems. For people who received standard chemotherapy, those with high disease burden were less likely than those with low disease burden to achieve remission. Further information in a plain language format is available in Supplementary Information (SI) Fig. S1.

## Introduction

Adults with relapsed or refractory (R/R) acute lymphoblastic leukemia (ALL) face a poor prognosis. With salvage chemotherapy, complete remission (CR) rates range from 18 to 44%, with median overall survival (OS) approximately 3–6 months^1–4^. Particularly for older adults, hematopoietic stem cell transplantation (HSCT) remains the established, potentially curative treatment; however, remission is typically required for HSCT to proceed. Improved therapies are therefore needed to increase remission rates, bridge patients to transplant, and improve survival in adults with R/R ALL.

Outcomes may be even poorer among R/R ALL patients with high disease burden^5,6^. Although there is no agreed measure of disease burden, some trials define high disease burden as bone marrow blast percentage (BMB) ≥50%, which is common among patients with R/R disease^5,7^. High BMB% has been associated with poor efficacy^5,6,8,9^ and safety outcomes^6,10^. The presence of extramedullary disease (EMD) can also be a surrogate for disease burden, and has been associated with poor efficacy outcomes^8^. Thus, there is an even greater need for more effective therapies to improve outcomes among adult patients with R/R ALL and high disease burden.

Inotuzumab ozogamicin (InO) is a calicheamicin-conjugated antibody targeting CD22 on B-cell ALL cells, and is approved for R/R B-cell precursor ALL^11,12^. In the phase 3 randomized INO-VATE trial, patients with R/R ALL had improved CR/CR with incomplete hematologic recovery (CRi) rates in the InO vs. SC arm, irrespective of disease burden (<50% BMB: 86.7% vs. 41.4%, ≥50% BMB: 77.9% vs. 24.4%)^7^. Similarly, a phase 2 trial demonstrated achievement of minimal residual disease (MRD) negativity in patients with various BMB%^13^. It is not known whether outcomes differ for patients with a higher BMB% (>90%), or whether these findings on CR/CRi rate and MRD negativity translate to other endpoints, including HSCT rate, OS, progression-free survival (PFS), and safety profile. It is also not known whether outcomes differ for patients with EMD or lymphoblastic lymphoma (LBL).

In this post hoc analysis of INO-VATE, our focus was to determine whether InO treatment remains efficacious and retains a similar safety profile for adult patients with R/R ALL with high baseline disease burden (defined herein as BMB > 90%). We also endeavored to determine whether CR/CRi rate and MRD negativity improvements affect other endpoints, including HSCT rate, PFS, OS, and safety profile. We report efficacy and safety outcomes for adult patients with R/R ALL by low, moderate, or high disease burden subgroup, as defined by BMB%. We also present additional analyses by baseline peripheral blast count and for patients with EMD/LBL. We report results of the primary endpoints of CR/CRi and OS, as well as secondary outcomes, including MRD negativity, HSCT rate, PFS, and adverse events (AEs). Data presented are from the final study database (through to January 4, 2017).

## Methods

### Patients and treatment

The global, randomized, open-label phase 3 INO-VATE study recruited 326 adult patients (aged ≥ 18 years) with CD22-positive B-cell ALL in first or second salvage. Patients could be Philadelphia-chromosome positive or negative. Patients who had ALL with EMD (and ≥5% BMB) were eligible to enroll, although patients with isolated extramedullary relapse were not eligible. Patients with documented LBL and ≥5% BMB at baseline assessment were also included. Patients were stratified by age (<55 vs. ≥55 years), salvage status (first or second salvage), and duration of first remission (<12 vs. ≥12 months). Patients were then randomized 1:1, using randomly permuted blocks within strata, to receive InO (*n* = 164) or SC (*n* = 162) treatment. Patient disposition and sample size determination were previously published^7,14^.

Patient BMB% was determined locally by morphologic assessment of bone marrow aspirate (or biopsy if clinically indicated). Patients with BMB% <50%, 50–90%, or >90% were categorized as having a low, moderate, or high disease burden, respectively. Patients with ALL and suspected or known EMD underwent imaging (e.g., magnetic resonance imaging or computerized tomography) to confirm EMD status before commencing study therapy and again during assessment of response. Baseline imaging was not required for patients with LBL, or for patients not suspected of having EMD. Patients with central nervous system involvement or an elevated peripheral absolute blast count ≥10 000/µL on the day of randomization were not enrolled (within 2 weeks of randomization, treatment to lower white blood cell count to below 10 000/µL, including steroids, hydroxyurea, or vincristine, was permitted). Patients who developed central nervous system disease during the study were assessed as having disease progression and treated in accordance with the investigator’s discretion.

Patients in the InO arm received 1.8 mg/m^2^ of InO intravenously each cycle, for a maximum of 6 cycles. Cycles lasted 21 days (cycle 1) or 28 days (subsequent cycles). Patients received 0.8 mg/m^2^ on day 1 and 0.5 mg/m^2^ on days 8 and 15. The day 1 dosage was reduced to 0.5 mg/m^2^ in patients achieving CR/CRi. Patients in the SC arm received the investigator’s choice of chemotherapy, including FLAG (fludarabine, cytarabine, and granulocyte colony-stimulating factor [GCSF]), cytarabine plus mitoxantrone, or high-dose cytarabine. Details of SC arm dosing regimens are provided in Supplementary Information (SI) Methods. Additional methodological details, including dose modifications, criteria for discontinuation, and patient monitoring schedules, have been previously published^7^.

The INO-VATE study is registered on ClinicalTrials.gov, NCT01564784. All patients provided written, informed consent, in accordance with the Declaration of Helsinki. The protocol was approved by an institutional review board and/or independent ethics committee at each trial center. All patients discontinued from the study by the last patient/last visit date of January 4, 2017. All authors had full access to all study data.

### Efficacy

Efficacy endpoints are reported in the intent-to-treat population, which consists of all randomized patients. The primary efficacy endpoints were OS and CR/CRi rate. OS was measured from the date of randomization to the date of death from any cause. Patients were followed for OS for up to 5 years or up to 2 years following randomization of the last patient (whichever occurred first). CR was defined as: <5% BMB, no leukemic blasts in peripheral blood, absolute neutrophil count ≥1000/μL, platelet count ≥100 000/μL, and the absence of EMD. The definition of CRi was the same as that for CR except with absolute neutrophil count <1000/μL and/or platelet count <100 000/μL. The secondary efficacy endpoints were PFS, MRD negativity, duration of remission (DoR), and HSCT rate, with definitions as previously described^7^.

Outcomes are reported according to the low, moderate, and high BMB% categories described above. CR/CRi and MRD negativity rates are presented for patients with baseline peripheral blast counts of 0/µL, >0 to 1000/µL, or >1000/µL. Efficacy outcomes are also presented for a group of patients who had either ALL with EMD at baseline or documented LBL. SAS statistical software (v9.1 or later; SAS Institute, Cary, NC) was used for all calculations. OS and PFS were calculated using Kaplan–Meier methods. Unstratified hazard ratio (HR) and 97.5% confidence interval (CI) were calculated using the Cox proportional hazard model. *P*-values were calculated using a one-sided unstratified log-rank test.

### Safety

Safety outcomes are reported for the safety population (all patients who received one or more dose of study drug) and grouped according to BMB% subgroups. AEs were categorized using the Medical Dictionary for Regulatory Activities (MedDRA) coding dictionary v19.1, and graded in accordance with the National Cancer Institute Common Terminology Criteria for Adverse Events (NCI CTCAE), v3.0. Treatment-emergent AEs (TEAEs) included AEs that occurred on or after the first dose but within 42 days of the last dose, and all subsequent treatment-related AEs. All veno-occlusive disease/sinusoidal obstruction syndrome (VOD/SOS) events within 2 years of the date of randomization were included, irrespective of causal attribution to study treatment.

## Results

### Patients and treatment

Subgroup size and patient demographic and disease characteristics were well balanced between treatment arms (SI Table S1). In the InO vs. SC arm, 53 vs. 48, 79 vs. 83, and 30 vs. 30 patients had a BMB% < 50%, 50–90%, and >90%, respectively. The median (range) BMB% was 26% (5–48%), 78% (50–90%), and 95% (91–100%) in the low, moderate, and high disease burden subgroups, respectively. Among patients in the InO vs. SC arm, baseline peripheral blast counts were 0/µL, >0–1000/µL, and >1000/µL in 71 vs. 74, 37 vs. 44, and 55 vs. 41 patients, respectively. At baseline, R/R ALL with EMD was identified in seven vs. five InO-arm vs. SC-arm patients; 11 and six patients, respectively, had documented LBL. For patients who had ALL with EMD, the most common tumor location was the lymph nodes (SI Table S2). Six non-nodal disease cases were identified.

### Efficacy

Remission rates in the InO arm were significantly higher than those in the SC arm and were similar in all categories of disease burden, whereas response rates in the SC arm declined with increasing disease burden. In the InO vs. SC arm, 73.6% vs. 45.8% (*p* = 0.0022), 74.7% vs. 26.5% (*p* < 0.0001), and 70.0% vs. 16.7% (*p* < 0.0001) of patients with low, moderate, and high BMB%, respectively, achieved CR/CRi (Table 1). Response rates were significantly lower in the high vs. low disease burden subgroup in the SC arm (*p* = 0.0042), but not in the InO arm (*p* = 0.3630).

**Table 1:** Efficacy outcomes by disease burden.

	BMB < 50%		BMB 50–90%		BMB > 90%	
	InO (*n* = 53)	SC (*n* = 48)	InO (*n* = 79)	SC (*n* = 83)	InO (*n* = 30)	SC (*n* = 30)
CR/CRi, *n* (%) [95% CI]	39 (73.6)	22 (45.8)	59 (74.7)	22 (26.5)	21 (70.0)	5 (16.7)
	[59.7–84.7]	[31.4–60.8]	[63.6–83.8]	[17.4–37.3]	[50.6–85.3]	[5.6–34.7]
Rate difference (97.5% CI), %	28 (7–49)		48 (33–64)		53 (29–78)	
* P*-value	0.0022		<0.0001		<0.0001	
CR, *n* (%) [95% CI]	17 (32.1)	8 (16.7)	30 (38.0)	14 (16.9)	8 (26.7)	3 (10.0)
	[19.9–46.3]	[7.5–30.2]	[27.3–49.6]	[9.5–26.7]	[12.3–45.9]	[2.1–26.5]
Rate difference (97.5% CI), %	15 (–3 to 34)		21 (6 to 36)		17 (–5 to 39)	
* P*-value	0.0366		0.0013		0.0903	
CRi, *n* (%) [95% CI]	22 (41.5)	14 (29.2)	29 (36.7)	8 (9.6)	13 (43.3)	2 (6.7)
	[28.1–55.9]	[17.0–44.1]	[26.1–48.3]	[4.3–18.1]	[25.5–62.6]	[0.8–22.1]
Rate difference (97.5% CI), %	12 (–9 to 34)		27 (13 to 41)		37 (14 to 59)	
* P*-value	0.0979		<0.0001		0.0011	
OS, median (95% CI), mo	7.4	9.1	7.7	7.1	7.3	3.9
	(5.2–11.5)	(4.6–13.8)	(5.8–10.8)	(4.9–9.4)	(4.7–11.8)	(2.1–5.0)
HR (97.5% CI)	0.643 (0.385–1.074)		0.806 (0.542–1.198)		0.601 (0.320–1.129)	
* P-*value	0.0260		0.1109		0.0335	
PFS, median (95% CI), mo	5.4	2.3	5	1.8	3.6	1.3
	(3.4–7.4)	(1.4–2.9)	(3.5–6.0)	(1.4–2.3)	(2.2–6.2)	(0.8–2.1)
HR (97.5% CI)	0.439 (0.262–0.735)		0.502 (0.336–0.750)		0.332 (0.161–0.686)	
* P*-value	0.0001		<0.0001		0.0002	
MRD negativity, *n*/*N*^*a*^ (%) [95% CI]	28/39 (71.8)	8/22 (36.4)	48/59 (81.4)	9/22 (40.9)	16/21 (76.2)	2/5 (40.0)
	[55.1–85.0]	[17.2–59.3]	[69.1–90.3]	[20.7–63.6]	[52.8–91.8]	[5.3–85.3]
* P*-value	0.0034		0.0002		0.1503	
Subsequent HSCT at any time, *n* (%) [95% CI]	27 (50.9)	13 (27.1)	38 (48.1)	22 (26.5)	14 (46.7)	1 (3.3)
	[36.8–64.9]	[15.3–41.8]	[36.7–59.6]	[17.4–37.3]	[28.3–65.7]	[0.1–17.2]

Data represent the intent-to-treat population.

*BMB* bone marrow blast, *CI* confidence interval, *CR/CRi* complete remission/complete remission with incomplete hematologic recovery, *HR* hazard ratio, *HSCT* hematopoietic stem cell transplantation, *MRD* minimal residual disease, *OS* overall survival, *PFS* progression-free survival.

^a^*N* = number of patients achieving CR/CRi in each treatment arm in the respective BMB subgroup.

MRD negativity among patients achieving CR/CRi was also higher in the InO arm compared with the SC arm, and did not appear to vary with disease burden. Among patients who achieved CR/CRi, MRD negativity was achieved by 71.8% (*n* = 28/39) vs. 36.4% (*n* = 8/22), 81.4% (*n* = 48/59) vs. 40.9% (*n* = 9/22), and 76.2% (*n* = 16/21) vs. 40.0% (*n* = 2/5) of InO-arm vs. SC-arm patients in the low, moderate, and high disease burden subgroups, respectively (Table 1). Similar results were obtained when patients were categorized by baseline peripheral blast count, with significantly higher CR/CRi rates shown among InO-arm vs. SC-arm patients with peripheral blast counts of 0/µL, >0–1000/µL, and >1000/µL, respectively: 87.3% (*n* = 62/71) vs. 45.9% (*n* = 34/74), *p* < 0.0001; 59.5% (*n* = 22/37) vs. 18.2% (*n* = 8/44), *p* < 0.0001; and 65.5% (*n* = 36/55) vs. 19.5% (*n* = 8/41), *p* < 0.0001. MRD negativity was achieved by 85.5% (*n* = 53/62) vs. 35.3% (*n* = 12/34), 68.2% (*n* = 15/22) vs. 50.0% (*n* = 4/8), and 66.7% (*n* = 24/36) vs. 37.5% (*n* = 3/8) of patients in the InO vs. SC arms with peripheral blast counts of 0/µL, >0–1000/µL, and >1000/µL, respectively.

In the InO arm, the post-treatment HSCT rate appeared to be independent of disease burden, whereas in the SC arm, the HSCT rate appeared lower in the high disease burden subgroup. Significantly more patients in the InO vs. SC arm proceeded to post-treatment HSCT across all categories of disease burden: 50.9% vs. 27.1% (*p* = 0.0072), 48.1% vs. 26.5% (*p* = 0.0022), and 46.7% vs. 3.3% (*p* < 0.0001) in the low, moderate, and high BMB% subgroups, respectively. Most of these patients were in remission before transplant, with 88.9% (*n* = 24/27) vs. 72.7% (*n* = 8/11), 89.5% (*n* = 34/38) vs. 63.6% (*n* = 14/22), and 64.3% (*n* = 9/14) vs. 100% (*n* = 1/1) of InO-arm vs. SC-arm patients in CR/CRi at last assessment before HSCT. Generally, patients who appeared most likely to benefit with improved OS were patients in the InO arm with low or moderate disease burden who proceeded to HSCT after achieving remission (OS HR [received vs. did not receive HSCT]: 0.384, *p* = 0.0065; 0.338, *p* = 0.0001; and 1.057, *p* = 0.5461 for InO-arm patients with low, moderate, and high disease burden, respectively, SI Fig. S2).

Among patients who achieved CR/CRi (including those who did and those who did not proceed to HSCT), DoR was generally longer in the InO vs. SC arm, and appeared longer in the low disease burden subgroup in both treatment arms. For InO-arm vs. SC-arm patients, the median DoR (95% CI, months) was 5.9 (4.3–8.8) vs. 3.6 (0.6–5.8), 5.3 (3.8–8.0) vs. 5.1 (2.7–12.3), and 4.9 (2.2–7.3) vs. 1.6 (1.0–3.1) for low, moderate, and high disease burden, respectively, and HRs (97.5% CI) were 0.379 (0.191–0.753), *p* = 0.0005; 0.994 (0.500–1.975), *p* = 0.4919; and 0.193 (0.042–0.884), *p* = 0.0035. Relapse rates in both treatment arms appeared higher in the high disease burden subgroup and lower in the InO vs. SC arm (33.3% [*n* = 13/39] vs. 86.4% [*n* = 19/22], 52.5% [*n* = 31/59] vs. 77.3% [*n* = 17/22], and 71.4% [*n* = 15/21] vs. 100% [*n* = 5/5] in the low, moderate, and high disease burden subgroups, respectively). For patients achieving CR/CRi, there were fewer deaths in remission in the high disease burden subgroup (InO vs. SC, *n*): 15/39 vs. 3/22, 15/59 vs. 3/22, and 3/21 vs. 0/5 for low, moderate, and high disease burden, respectively.

**Fig. 1:**
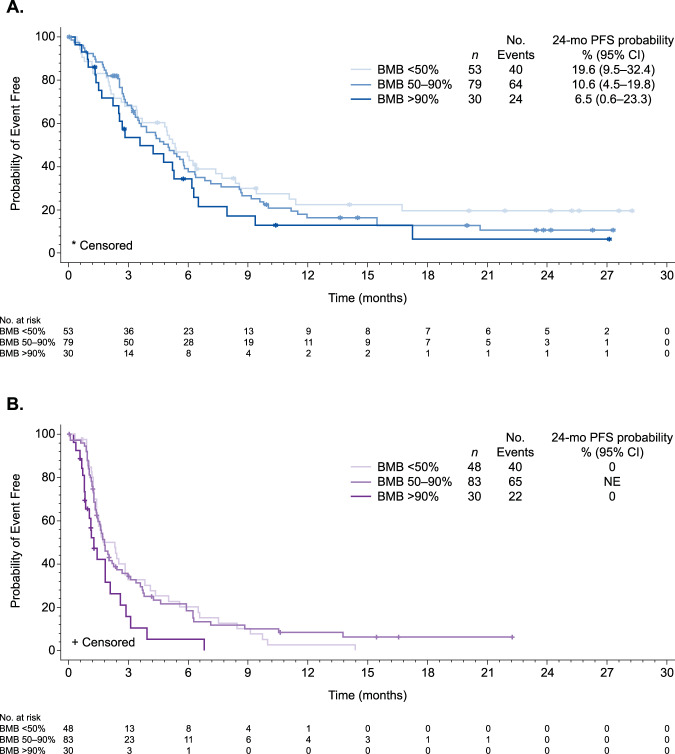
Progression-free survival by bone marrow status. Kaplan–Meier plots show progression-free survival by baseline bone marrow blasts (<50%, 50–90%, and >90%) for patients in the (**a**) InO arm and (**b**) SC arm. *BMB* bone marrow blasts, *InO* inotuzumab ozogamicin, *PFS* progression-free survival, *SC* standard of care chemotherapy.

For all categories of disease burden, patients in the InO vs. SC arm had significantly improved PFS (HR [97.5% CI] 0.44 [0.26–0.74], *p* = 0.0001; 0.50 [0.34–0.75], *p* < 0.0001; and 0.33 [0.16–0.69], *p* = 0.0002 for low, moderate, and high BMB%, respectively). The probability of 24-month PFS in the InO arm appeared to decline with greater disease burden (19.6%, 10.6%, and 6.5% for low, moderate, and high disease burden, respectively), and was either zero or not estimable (though ≤ 6.2%) in all subgroups in the SC arm (Fig. 1).

**Fig. 2:**
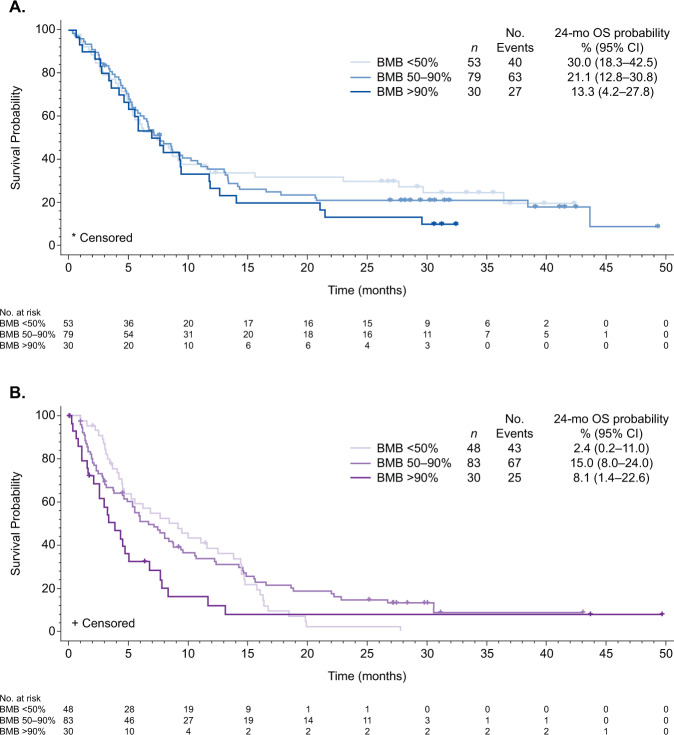
Overall survival by bone marrow status. Kaplan–Meier plots show overall survival by baseline bone marrow blasts (<50%, 50–90%, and >90%) for patients in the (**a**) InO arm and (**b**) SC arm. *BMB* bone marrow blasts, *InO* inotuzumab ozogamicin, *OS* overall survival, *SC* standard of care chemotherapy.

There was a trend towards improved OS in the InO vs. SC arm: HR (97.5% CI) 0.64 (0.39–1.07), *p* = 0.0260; 0.81 (0.54–1.20), *p* = 0.1109; and 0.60 (0.32–1.13), *p* = 0.0335, whereas median OS (95% CI, months) was 7.4 (5.2–11.5) vs. 9.1 (4.6–13.8), 7.7 (5.8–10.8) vs. 7.1 (4.9–9.4), and 7.3 (4.7–11.8) vs. 3.9 (2.1–5.0) for low, moderate, and high BMB%, respectively. The probability of 24-month OS was higher with InO vs. SC across all three subgroups, with a trend towards improved outcomes with lower disease burden in the InO arm (Fig. 2).

In terms of response rates, InO vs. SC treatment also appeared to benefit patients with EMD/LBL, although the sample sizes were small. The CR/CRi rate was significantly higher with InO vs. SC (66.7% [*n* = 12/18] vs. 18.2% [*n* = 2/11], *p* = 0.0144), with a median DoR (95% CI, months) of 5.2 (1.8–not evaluable) vs. 5.4 (2.9–8.0); and HR 0.829, *p* = 0.4042. Among those achieving CR/CRi, *n* = 7/12 vs. *n* = 1/2 InO-arm vs. SC-arm patients were MRD-negative. The OS HR was 0.661 (97.5% CI, 0.269–1.621, *p* = 0.1478), with a median OS (95% CI) for InO vs. SC of 5.9 (3.4–9.4) vs. 5.5 (2.1–6.7) months. Concurrently, the median PFS (95% CI) was 4.4 (1.9–7.1) vs. 1.6 (0.8–3.7) months, with HR 0.502 (97.5% CI, 0.203–1.240, *p* = 0.0410). Among patients with baseline EMD, five of seven in the InO arm and two of five in the SC arm achieved CR/CRi, which includes resolution of EMD. For patients with documented LBL, *n* = 7/11 in the InO arm and *n* = 0/6 in the SC arm achieved CR/CRi. Among patients in the InO vs. SC arm, *n* = 4/59 vs. *n* = 2/41 relapses involved the development of new EMD.

### Safety

Dose reductions and temporary or permanent discontinuations due to TEAEs were either equally common or more common with InO vs. SC irrespective of disease burden (SI Table S3). With InO, infections and infestations were the leading reasons for permanent discontinuation irrespective of disease burden. Compared with infections and infestations, hepatobiliary disorders were equally common reasons for permanent discontinuation in the low disease burden subgroup. Blood and lymphatic system disorders were the leading reasons for temporary discontinuation (SI Table S3).

Irrespective of treatment arm or disease burden, the most frequent all-grade and grade ≥3 TEAEs were hematologic (Table 2 and SI Table S4). The incidence of febrile neutropenia with InO treatment appeared to increase with increasing disease burden (17.0%, 21.5%, and 53.3% for low, moderate, and high disease burden, respectively), whereas with SC treatment, febrile neutropenia remained high for all disease burden subgroups (55.8%, 54.9%, and 46.4%). The rates of other hematologic TEAEs did not appear to vary substantially with disease burden (Table 2). Grade ≥3 hematologic laboratory abnormalities, including decreased hemoglobin, leukocytes, neutrophil and platelet counts, and lymphopenia, appeared to increase with increasing disease burden in the InO arm, but did not appear to vary with disease burden in the SC arm (Table 2).

**Table 2:** Treatment-emergent adverse events and hematologic laboratory abnormalities.

Event, *n* (%)	BMB < 50%		BMB 50–90%		BMB > 90%	
	InO (*n* = 53)	SC (*n* = 43)	InO (*n* = 79)	SC (*n* = 71)	InO (*n* = 30)	SC (*n* = 28)
**TEAEs (grade≥3)**^**a**^						
Any TEAE	47 (88.7)	42 (97.7)	71 (89.9)	69 (97.2)	29 (96.7)	26 (92.9)
Blood and lymphatic system disorders	41 (77.4)	38 (88.4)	62 (78.5)	63 (88.7)	26 (86.7)	22 (78.6)
Thrombocytopenia	21 (39.6)	29 (67.4)	36 (45.6)	42 (59.2)	10 (33.3)	14 (50.0)
Neutropenia	23 (43.4)	18 (41.9)	44 (55.7)	36 (50.7)	9 (30.0)	8 (28.6)
Anemia	10 (18.9)	22 (51.2)	18 (22.8)	29 (40.8)	8 (26.7)	11 (39.3)
Leukopenia	9 (17.0)	19 (44.2)	24 (30.4)	28 (39.4)	11 (36.7)	6 (21.4)
Lymphopenia	9 (17.0)	15 (34.9)	12 (15.2)	17 (23.9)	5 (16.7)	4 (14.3)
Febrile neutropenia	9 (17.0)	24 (55.8)	17 (21.5)	39 (54.9)	16 (53.3)	13 (46.4)
Hepatobiliary disorders	11 (20.8)	3 (7.0)	13 (16.5)	6 (8.5)	4 (13.3)	3 (10.7)
VOD/SOS^b^	7 (13.2)	1 (2.3)	10 (12.7)	2 (2.8)	2 (6.7)	0
Hyperbilirubinemia	4 (7.5)	2 (4.7)	3 (3.8)	4 (5.6)	3 (10.0)	3 (10.7)
Infections and infestations	18 (34.0)	25 (58.1)	18 (22.8)	38 (53.5)	12 (40.0)	15 (53.6)
Bacteremia	2 (3.8)	4 (9.3)	1 (1.3)	5 (7.0)	3 (10.0)	1 (3.6)
Neutropenic sepsis	0	1 (2.3)	2 (2.5)	3 (4.2)	3 (10.0)	2 (7.1)
Klebsiella bacteremia	1 (1.9)	1 (2.3)	0	1 (1.4)	0	3 (10.7)
Sepsis	1 (1.9)	3 (7.0)	3 (3.8)	6 (8.5)	1 (3.3)	3 (10.7)
Investigations	18 (34.0)	10 (23.3)	23 (29.1)	14 (19.7)	8 (26.7)	9 (32.1)
GGT increased	8 (15.1)	4 (9.3)	8 (10.1)	2 (2.8)	2 (6.7)	1 (3.6)
AST increased	2 (3.8)	1 (2.3)	2 (2.5)	2 (2.8)	3 (10.0)	2 (7.1)
ALT increased	1 (1.9)	1 (2.3)	3 (3.8)	3 (4.2)	2 (6.7)	3 (10.7)
Metabolism and nutrition disorders	9 (17.0)	8 (18.6)	12 (15.2)	16 (22.5)	6 (20.0)	7 (25.0)
Hypokalemia	4 (7.5)	2 (4.7)	6 (7.6)	10 (14.1)	1 (3.3)	1 (3.6)
Hypocalcemia	0	0	2 (2.5)	1 (1.4)	1 (3.3)	4 (14.3)
**Hematologic laboratory abnormalities (grade≥3)**						
Activated partial thromboplastin time prolonged	0	1 (2.3)	2 (2.5)	2 (2.8)	3 (10.0)	0
Hemoglobin decreased	17 (32.1)	27 (62.8)	27 (34.2)	54 (76.1)	23 (76.7)	20 (71.4)
INR increased	0	0	1 (1.3)	1 (1.4)	0	0
Leukocytes decreased	36 (67.9)	42 (97.7)	66 (83.5)	70 (98.6)	29 (96.7)	27 (96.4)
Lymphopenia	32 (60.4)	38 (88.4)	56 (70.9)	58 (81.7)	26 (86.7)	21 (75.0)
Neutrophil count decreased	38 (71.7)	36 (83.7)	71 (89.9)	56 (78.9)	29 (96.7)	22 (78.6)
Platelet count decreased	34 (64.2)	42 (97.7)	60 (75.9)	71 (100.0)	29 (96.7)	27 (96.4)
Prothrombin time increased	0	1 (2.3)	0	0	0	0

Data represent the safety population.

TEAEs and hematologic laboratory abnormalities were graded according to the NCI CTCAE, version 3.0.

*ALT* alanine aminotransferase, *AST* aspartate aminotransferase, *BMB* bone marrow blast, *GGT* gamma-glutamyl transferase, *InO* inotuzumab ozogamacin, *INR* international normalized ratio, *NCI CTCAE* National Cancer Institute Common Terminology Criteria for Adverse Events, *SC* standard of care chemotherapy, *TEAE* treatment-emergent adverse event, *VOD/SOS* veno-occlusive disease/sinusoidal obstruction syndrome.

^a^All-causality TEAEs grade ≥3 with ≥10% incidence occurring in either arm (any treatment cycle, any BMB subgroup) are shown.

^b^In July 2017 (after the clinical database was locked), a fourth case of VOD/SOS was confirmed to have occurred in an SC arm patient. This case of VOD/SOS occurred in March 2013, was not entered on the clinical report form, and is therefore not included.

Hepatotoxicity TEAEs were more common with InO vs. SC irrespective of disease burden, with a possible reduction in VOD/SOS rate in the high disease burden subgroup. With InO vs. SC, grade ≥3 VOD/SOS was reported in 13.2 vs. 2.3%, 12.7 vs. 2.8%, and 6.7 vs. 0% of patients with low, moderate, and high disease burden, respectively (Table 2). Concurrently, post-HSCT VOD/SOS was reported in 25.9% (*n* = 7/27) vs. 7.7% (*n* = 1/13), 23.7% (*n* = 9/38) vs. 9.5% (*n* = 2/21), and 14.3% (*n* = 2/14) vs. 0% (*n* = 0/1) of patients.

Grade 5 TEAE incidence with InO treatment appeared to decline with increasing disease burden, whereas with SC, grade 5 TEAE incidence appeared to increase with increasing disease burden. Grade 5 TEAEs with InO vs. SC were reported in 22.6% (*n* = 12/53) vs. 7.0% (*n* = 3/43), 13.9% (*n* = 11/79) vs. 9.9% (*n* = 7/71), and 10.0% (*n* = 3/30) vs. 21.4% (*n* = 6/28) of patients with low, moderate, and high disease burden, respectively. There were no notable differences in TEAEs for patients with EMD/LBL compared with the overall analysis population.

## Discussion

This post hoc analysis of the phase 3 INO-VATE trial confirms and extends previous reports of improved outcomes with InO compared with SC treatment^7,14^. Our analysis confirmed that InO remains efficacious and has a similar safety profile for R/R ALL in patients with a high disease burden. It also showed that reports of improved remission and MRD-negativity rates with InO in different disease burden subgroups^7,14^ extend to selected other endpoints. Moreover, the similar safety profile with InO for all disease burden subgroups, and its effectiveness in individual cases of EMD/LBL, are clinically relevant and support the use of InO in these challenging patient subpopulations.

Patients in the InO vs. SC arm had significantly improved CR/CRi rate, PFS, and HSCT rate in all three disease burden subgroups, with high (≥70%) CR/CRi rates with InO irrespective of BMB%. By contrast, CR/CRi rates in the SC arm were significantly lower for patients with high vs. low disease burden. In addition, most patients in the InO arm achieved CR/CRi irrespective of CD22 expression level (65.7 vs. 78.5% for patients with <90% vs. ≥90% leukemic blast positivity, respectively)^14^. CR/CRi rates also appeared higher in the InO vs. SC arm irrespective of salvage treatment phase, although the small sample sizes within disease burden subgroups precluded a robust comparison (data not shown). These high remission rates are clinically important since remission is required for proceeding to HSCT. Although some individuals <25 years of age have achieved long-term remission (≤39 months) with chimeric antigen receptor T cell (CAR T) therapy^15,16^, HSCT remains the established potentially curative treatment option, particularly for older adults with R/R ALL. The robust remission rate in the high disease burden subgroup of INO-VATE contrasts with another targeted treatment, blinatumomab, where remission rates were lower in patients with ≥50% BMB vs. <50% BMB (29 vs. 73%)^5^. Similarly, in another study, patients with higher BMB% (≥5% vs. <5%) treated with CAR T cell therapy experienced poorer outcomes (remission rate 75 vs. 95%, median OS 12.4 vs. 20.1 months)^6^. This may be due to limitations in number of CAR T cells, because high disease burden patients require more CAR T cells to create an effective ratio of CAR T cells to disease burden.^6^

The improved CR/CRi rate seen with InO vs. SC, irrespective of disease burden, was consistent with improvements in other efficacy endpoints. For all BMB subgroups, the MRD negativity rate and the 24-month OS and PFS probabilities were higher in InO-arm vs. SC-arm patients. The high MRD negativity rates observed across all subgroups are consistent with phase 2 results for InO^13^. This is clinically relevant since MRD negativity is a key prognostic factor for OS and post-transplant outcomes in the R/R setting, particularly in first salvage^17–19^. Despite these similar rates of CR/CRi and MRD negativity across BMB% subgroups in the InO arm, probabilities of 24-month OS and PFS appeared greater in the low BMB% subgroup, compared with moderate or high BMB%. This finding is consistent with the higher relapse rates observed in the high disease burden subgroup, and may suggest that additional factors besides disease burden have impacted on relapse rates and survival. Although small patient numbers limit the generalizability of results in patients with EMD/LBL, InO-arm vs. SC-arm patients with EMD/LBL showed a clinically important improvement in the remission rate, consistent with prior reports on the effectiveness of InO in patients with EMD^20^. This may be an advantage of InO treatment, since the presence or history of EMD may predict poor responses to other therapies, including blinatumomab^8^. Overall, these results across secondary endpoints and in patients with EMD/LBL further suggest that InO is superior to SC treatment, including patients with high disease burden.

Increased baseline disease burden did not negatively impact the safety profile of InO. Despite increased treatment cycles among InO-treated patients, overall TEAE incidence was similar between treatment arms. However, dose reductions and temporary and permanent discontinuations due to TEAEs were more common with InO vs. SC. Consistent with prior findings, the most common TEAEs were cytopenias^7,13^. The incidence of febrile neutropenia was lower with InO vs. SC in the low and moderate disease burden subgroups, but similar with InO or SC in the high disease burden subgroup. Similar to previous reports, hepatotoxicity was more common with InO vs. SC^7,13^. There were fewer deaths in remission in the high disease burden subgroup; and results suggested a possible reduction in VOD/SOS incidence among patients with high disease burden, which may suggest an impact of disease burden on InO exposure. Increased InO exposure has been associated with an increased risk of VOD/SOS following HSCT, leading to the recommendation that if proceeding to HSCT, InO exposure should be limited to two or fewer cycles (three cycles if necessary to achieve an MRD-negative CR/CRi)^11,12,21–23^. This is feasible considering that, in a prior report, most patients in the InO arm who achieved CR/CRi did so following the first cycle of treatment (73%)^7^. A clinical trial is currently underway to investigate lower doses of InO and the impact of dose on the benefit-to-risk ratio of InO (NCT03094611). InO exposure has not been found to alter VOD/SOS risk among patients not proceeding to HSCT^21,23^. Another factor found to be associated with post-HSCT VOD/SOS risk is pre-study HSCT^14^. In the INO-VATE trial, prior HSCT was associated with post-HSCT VOD/SOS in the InO arm (odds ratio 6.02; *p* = 0.032)^14^. In a combined analysis of two studies, InO-treated patients proceeding to HSCT had a lower non-relapse mortality rate and improved long-term survival if they had not previously received HSCT^22^. These results differ from a report on CAR T cell therapy where AEs, including cytokine release syndrome (CRS) and neurotoxicity, were more common among patients with high vs. low disease burden (≥5% vs. <5% BMB: 41% vs. 5% for severe CRS and 59% vs. 14% for severe neurotoxicity)^6^. CRS appears to be less common (≤6% incidence) in blinatumomab-treated patients, but has been reported in patients with high disease burden^5,9,24^. The lack of increase in AEs among InO-treated patients with high disease burden may be another advantage of InO treatment in this population.

This analysis has several limitations. Firstly, this is a post hoc subgroup analysis of INO-VATE and results should therefore be interpreted with caution. Also, patients with peripheral blasts ≥10 000/µL were excluded from the trial, which would have affected the enrolled patient population, and may impact the generalizability of these results. However, these impacts would be expected to affect both treatment arms equally, and InO provided substantial benefits compared with SC irrespective of baseline peripheral blast count or BMB%. BMB% was used as the primary measure of disease burden because it is a mandatory test for ALL diagnosis^25^ and has been similarly used in previous ALL studies^5–7,13^. Although confidence intervals would have been impacted by the differing sizes of the disease burden subgroups, the optimal BMB% thresholds were selected based on the patient distribution. The <50% threshold for low BMB% was derived from previous literature^5,7,13^, the moderate (50–90%) threshold was selected because the majority of patients had BMB ≥ 50%, and the high (>90%) threshold was added to allow an assessment of patients with the highest percentage of BMB while still allowing reasonable sample sizes. Consistent with previous reports from the INO-VATE trial^7^, some of the survival curves in this analysis appeared to deviate from the proportional hazards model. Despite this limitation, Kaplan–Meier curves and HRs for OS showed a trend towards improvement with InO vs. SC treatment in all BMB subgroups, with a greater survival benefit at later time points, consistent with the benefit of InO vs. SC based on other endpoints. Although the overall prognosis remains poor among InO-arm patients with high disease burden (13.3% OS at 24 months), InO still provides substantially improved outcomes compared with SC treatment, with significantly improved remission rates, HSCT rates, and PFS. These improved outcomes, in turn, may contribute to the lower hospitalization burden seen with InO compared with SC treatment^26^. The ability of InO to be delivered either in the inpatient or outpatient setting may also contribute to the positive benefit-to-risk ratio of InO treatment.

Our analysis indicated overall that, compared with the whole INO-VATE trial population, InO remains efficacious and retains a similar safety profile for R/R ALL patients in challenging subpopulations, including patients with high baseline disease burden. Our analysis extends previous reports of improved CR/CRi rate and MRD negativity among patients with various BMB% to additional endpoints, including HSCT rate and PFS. The safety profile of InO was similar for all disease burden subgroups, suggesting that high disease burden does not negatively impact the safety profile of InO. Patients with baseline EMD or LBL had similar efficacy and safety outcomes to patients without EMD or LBL. Potential directions for future research include investigating whether these positive results with InO in adult R/R ALL can be reproduced in the frontline setting, determining whether the benefits of InO could be further extended when administered in combination with other therapies, and examining the potential utility of InO in bridging patients with high disease burden to other novel therapies. In conclusion, this study supports the use of InO treatment across all baseline disease burden subgroups, including patients with high disease burden R/R ALL.

A plain language summary of this article is available in Supplementary Information (SI) Figure S1.

## Supplementary information

SI Figure S1: Plain language summary

SI Methods and Tables S1-S4

SI Fig. S2

## Data Availability

Upon request, and subject to certain criteria, conditions and exceptions (see https://www.pfizer.com/science/clinical-trials/trial-data-and-results for more information), Pfizer will provide access to individual deidentified participant data from Pfizer-sponsored global interventional clinical studies conducted for medicines, vaccines, and medical devices (1) for indications that have been approved in the United States and/or European Union, or (2) in programs that have been terminated (ie, development for all indications has been discontinued). Pfizer will also consider requests for the protocol, data dictionary, and statistical analysis plan. Data may be requested from Pfizer trials 24 months after study completion. The deidentified participant data will be made available to researchers whose proposals meet the research criteria and other conditions, and for which an exception does not apply, via a secure portal. To gain access, data requestors must enter into a data access agreement with Pfizer.

## References

[1] Gokbuget N (2012). Outcome of relapsed adult lymphoblastic leukemia depends on response to salvage chemotherapy, prognostic factors, and performance of stem cell transplantation. Blood.

[2] Oriol A (2010). Outcome after relapse of acute lymphoblastic leukemia in adult patients included in four consecutive risk-adapted trials by the PETHEMA Study Group. Haematologica.

[3] Tavernier E (2007). Outcome of treatment after first relapse in adults with acute lymphoblastic leukemia initially treated by the LALA-94 trial. Leukemia.

[4] O’Brien S (2008). Outcome of adults with acute lymphocytic leukemia after second salvage therapy. Cancer.

[5] Topp MS (2015). Safety and activity of blinatumomab for adult patients with relapsed or refractory B-precursor acute lymphoblastic leukaemia: a multicentre, single-arm, phase 2 study. Lancet Oncol..

[6] Park JH (2018). Long-term follow-up of CD19 CAR therapy in acute lymphoblastic leukemia. N. Engl. J. Med..

[7] Kantarjian HM (2016). Inotuzumab ozogamicin versus standard therapy for acute lymphoblastic leukemia. N. Engl. J. Med..

[8] Aldoss I (2017). Correlates of resistance and relapse during blinatumomab therapy for relapsed/refractory acute lymphoblastic leukemia. Am. J. Hematol..

[9] Kantarjian H (2017). Blinatumomab versus chemotherapy for advanced acute lymphoblastic leukemia. N. Engl. J. Med..

[10] Mueller KT (2017). Cellular kinetics of CTL019 in relapsed/refractory B-cell acute lymphoblastic leukemia and chronic lymphocytic leukemia. Blood.

[11] Pfizer Inc. BESPONSA® (inotuzumab ozogamicin) prescribing information. New York: Wyeth Pharmaceuticals; c2017 [last update Mar 2018]. http://labeling.pfizer.com/ShowLabeling.aspx?id=9503. Accessed Jan 2020.

[12] European Medicines Agency. BESPONSA® (inotuzumab ozogamicin) summary of product characteristics. London: EMA; c2017 [last update Nov 25, 2019]. https://www.ema.europa.eu/en/medicines/human/EPAR/besponsa. Accessed Jan 2020.

[13] DeAngelo DJ (2017). Inotuzumab ozogamicin in adults with relapsed or refractory CD22-positive acute lymphoblastic leukemia: a phase 1/2 study. Blood Adv..

[14] Kantarjian HM (2019). Inotuzumab ozogamicin versus standard of care in relapsed or refractory acute lymphoblastic leukemia: final report and long-term survival follow-up from the randomized, phase 3 INO-VATE study. Cancer.

[15] Grupp SA (2015). Durable remissions in children with relapsed/refractory ALL treated with T cells engineered with a CD19-targeted chimeric antigen receptor (CTL019). Blood.

[16] Maude SL (2016). Sustained remissions with CD19-specific chimeric antigen receptor (CAR)-modified T cells in children with relapsed/refractory ALL. J. Clin. Oncol..

[17] Jabbour E (2020). Impact of minimal residual disease status in patients with relapsed/refractory acute lymphoblastic leukemia treated with inotuzumab ozogamicin in the phase III INO-VATE trial. Leuk. Res..

[18] Jabbour E (2017). Differential impact of minimal residual disease negativity according to the salvage status in patients with relapsed/refractory B-cell acute lymphoblastic leukemia. Cancer.

[19] Eckert C (2013). Minimal residual disease after induction is the strongest predictor of prognosis in intermediate risk relapsed acute lymphoblastic leukaemia - long-term results of trial ALL-REZ BFM P95/96. Eur. J. Cancer.

[20] Bertamini L (2018). Inotuzumab ozogamicin is effective in relapsed/refractory extramedullary B acute lymphoblastic leukemia. BMC Cancer.

[21] Kantarjian HM (2017). Hepatic adverse event profile of inotuzumab ozogamicin in adult patients with relapsed or refractory acute lymphoblastic leukaemia: results from the open-label, randomised, phase 3 INO-VATE study. Lancet Haematol..

[22] Marks DI (2019). Outcomes of allogeneic stem cell transplantation after inotuzumab ozogamicin treatment for relapsed or refractory acute lymphoblastic leukemia. Biol. Blood Marrow Transplant.

[23] Kebriaei P (2018). Management of important adverse events associated with inotuzumab ozogamicin: expert panel review. Bone Marrow Transplant.

[24] Topp MS (2014). Phase II trial of the anti-CD19 bispecific T cell–engager blinatumomab shows hematologic and molecular remissions in patients with relapsed or refractory B-precursor acute lymphoblastic leukemia. J. Clin. Oncol..

[25] Hoelzer D (2016). Acute lymphoblastic leukaemia in adult patients: ESMO Clinical Practice Guidelines for diagnosis, treatment and follow-up. Ann. Oncol..

[26] Marks DI (2019). Burden of hospitalization in acute lymphoblastic leukemia patients treated with inotuzumab ozogamicin versus standard chemotherapy treatment. Cancer Med..

