# Supplementary material for: Inotuzumab ozogamicin for relapsed/refractory acute lymphoblastic leukemia: outcomes by disease burden
Source: Blood Cancer J. 2020 Aug 7;10(8):81. doi: 10.1038/s41408-020-00345-8 (PMC7414105; doi:10.1038/s41408-020-00345-8)
Supplement: Supplementary file 1 — SI Figure S1: Plain language summary [file 41408_2020_345_MOESM1_ESM.pdf]

SI Figure S1: Plain Language Summary

Please note that this summary only contains information from the full scientific article:
View Scientific Article

How does inotuzumab ozogamicin compare with standard chemotherapy for people with acute lymphoblastic leukemia who have different levels of cancer cells in their bone marrow?

Date of summary: June 2020

Study number: NCT01564784 | **Study start date:** August 2012 | **Study end date:** January 2017

The full title of this article is: Inotuzumab ozogamicin for relapsed/refractory acute lymphoblastic leukemia: outcomes by disease burden

The purpose of this plain language summary is to help you to understand the findings from recent research.

This study was sponsored by Pfizer.

Inotuzumab ozogamicin is approved to treat the condition under study that is discussed in this summary.

The results of this study may differ from those of other studies. Researchers should make treatment decisions based on all available evidence, not on the results of a single study.

More information can be found in the scientific article of this study, which you can access here:
View Scientific Article

Acute lymphoblastic leukemia <uh-KYOOT LIM-foh-BLAS-tik loo-KEE-mee-uh>

ALL <A-ell-ell>

Cytopenia <SY-toh-PEE-nee-uh>

Extramedullary <EK-struh-MED-yoo-LAYR-ee>

Febrile neutropenia <FEH-brile noo-troh-PEE-nee-uh>

Inotuzumab ozogamicin <ih-noh-TOO-zoo-mab OH-zoh-ga-MIH-sin>

Lymphoblast <LIM-foh-BLAST>

Lymphoblastic lymphoma <LIM-foh-BLAS-tik lim-FOH-muh>

What did this study look at?

- Acute lymphoblastic leukemia (ALL for short) is a type of blood cancer. In ALL, the body makes too many immature white blood cells called lymphoblasts.
  - These lymphoblast cells are normally found in the bone marrow. In people with ALL, lymphoblast cells may also be in the blood.
  - In some people with ALL, these lymphoblast cells can also form a tumor in a different part of the body. This is called extramedullary disease.
  - In some people with ALL, the cancer can become undetectable but then come back (known as relapsed ALL), or the cancer can stop responding to treatment (known as refractory ALL).
- Inotuzumab ozogamicin (InO for short) is a treatment for people with relapsed or refractory ALL (R/R ALL for short).
  - Most lymphoblast cancer cells have a protein called CD22 on their surface.
  - InO works by finding and destroying ALL cancer cells by binding to CD22.
- This study looked at people who received either InO or standard chemotherapy for R/R ALL.
  - Compared with people who received standard chemotherapy, people who received InO were more likely to:
    - Have no signs of their cancer (called remission)
    - Live longer.
- Researchers also looked at how people with low, medium, and high levels of lymphoblast cells in their bone marrow (called disease burden) responded to treatment with InO or chemotherapy.
  - This is important because people with a high disease burden are often sicker and do not live as long as people with a lower disease burden.
- In this summary, researchers divided people into 3 groups, based on whether they had a low, medium, or high level of lymphoblast cells in their bone marrow:
  - Low disease burden
  - Medium disease burden
  - High disease burden.
- Researchers compared InO with standard chemotherapy in terms of efficacy* and medical problems.**
  - Researchers wanted to know whether the way these treatments work is affected by:
    - A person's level of disease burden
    - Whether or not a person has extramedullary disease.

*Efficacy is how well a drug works within a clinical trial
**Medical problems could be caused by reasons not related to the study (for example, caused by an underlying disease or by chance). Or, medical problems could have been caused by a study treatment, or by another medicine the participant was taking.

Where is this study in the drug development timeline?

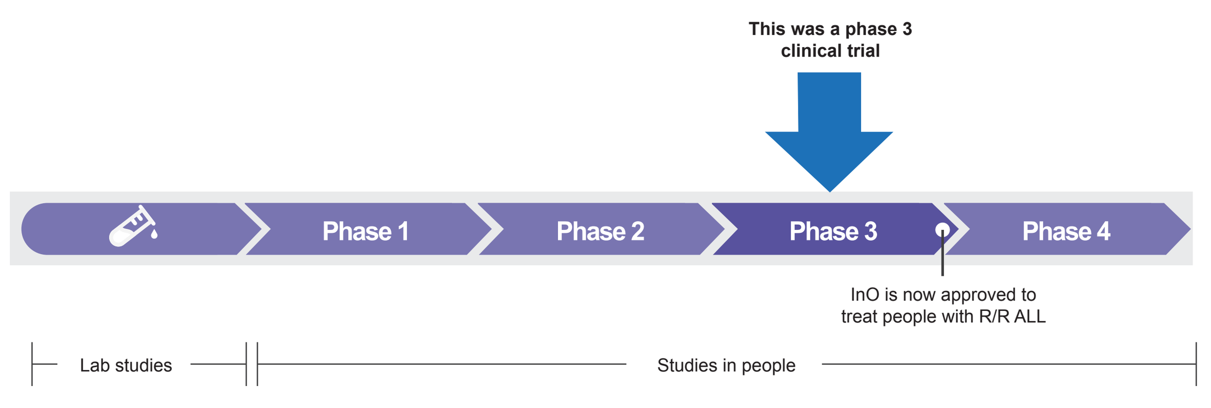

Who took part in this study?

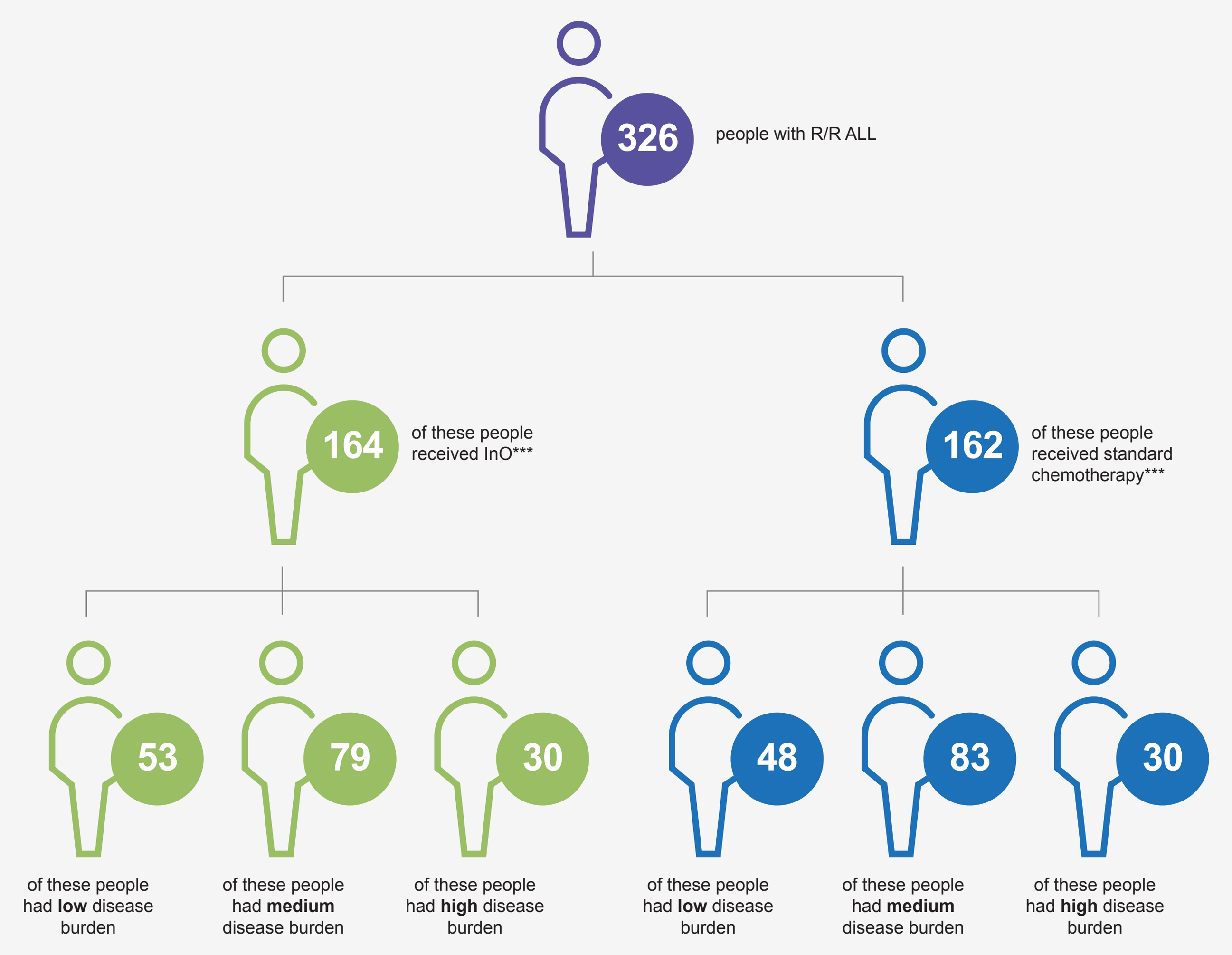

***The level of disease burden was not known for 2 people who received InO and 1 person who received standard chemotherapy

- The numbers above include 29 people who had extramedullary disease:
  - 18 received InO
  - 11 received standard chemotherapy.
- All of the people in this study had more than a certain minimum level of ALL cells in their bone marrow.
  - A small number of people had another, similar type of blood cancer called lymphoblastic lymphoma. These people were included in the extramedullary disease group.

What were the results of the study?

A higher proportion of people achieved remission with InO compared with standard chemotherapy across all groups

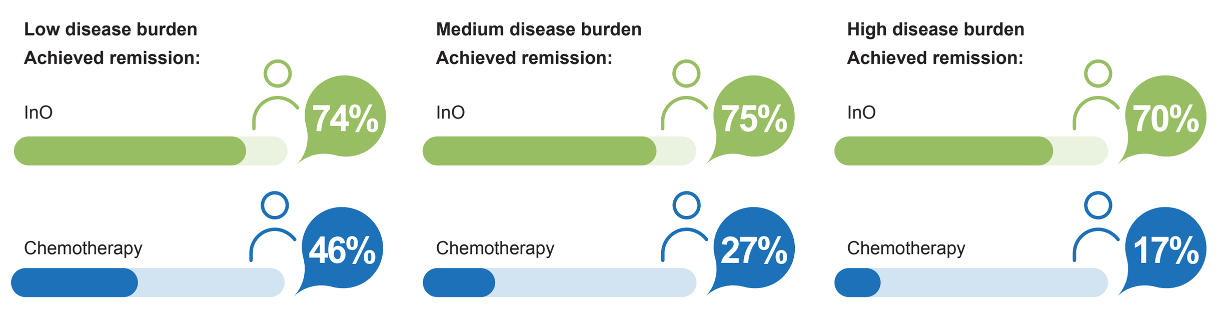

- For people who received InO, those with high disease burden were just as likely to achieve remission as those with low disease burden.
- For people who received standard chemotherapy, those with high disease burden were significantly less likely to achieve remission than those with low disease burden.

People who received InO were more likely to live to the end of the study than people who received standard chemotherapy

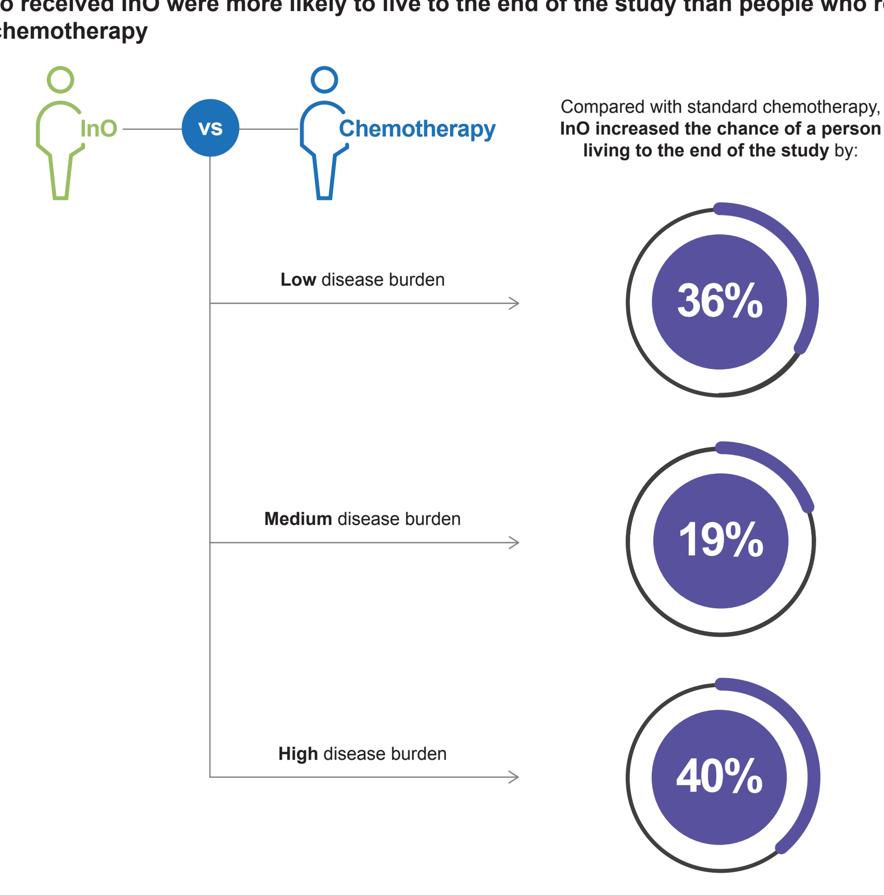

More people who received InO reached the end of the study without their cancer getting worse

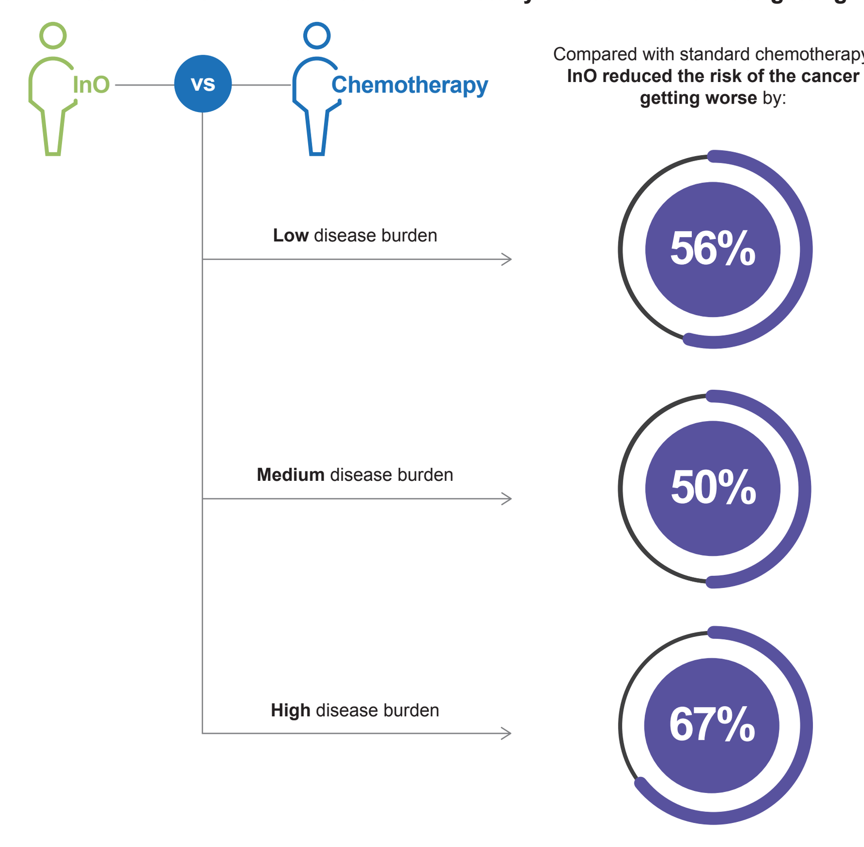

- For people with extramedullary disease:
  - 67% achieved remission with InO compared with 18% with standard chemotherapy
  - Compared with those who received standard chemotherapy, those who received InO were:
    - 34% more likely to live to the end of the study
    - 50% more likely to reach the end of the study without their cancer getting worse.
- Whether people received InO or standard chemotherapy, the most common severe medical problem**** was low numbers of certain blood cells (a condition called cytopenia).
  - Rates of cytopenia were similar for all disease burden groups (between 77 and 89 people in 100).

****A medical problem is considered "severe" when it limits daily activities such as bathing and dressing, is disabling or is medically significant, or could be life-threatening, need hospital care, or cause lasting problems.

The proportion of people who experienced certain severe medical problems:

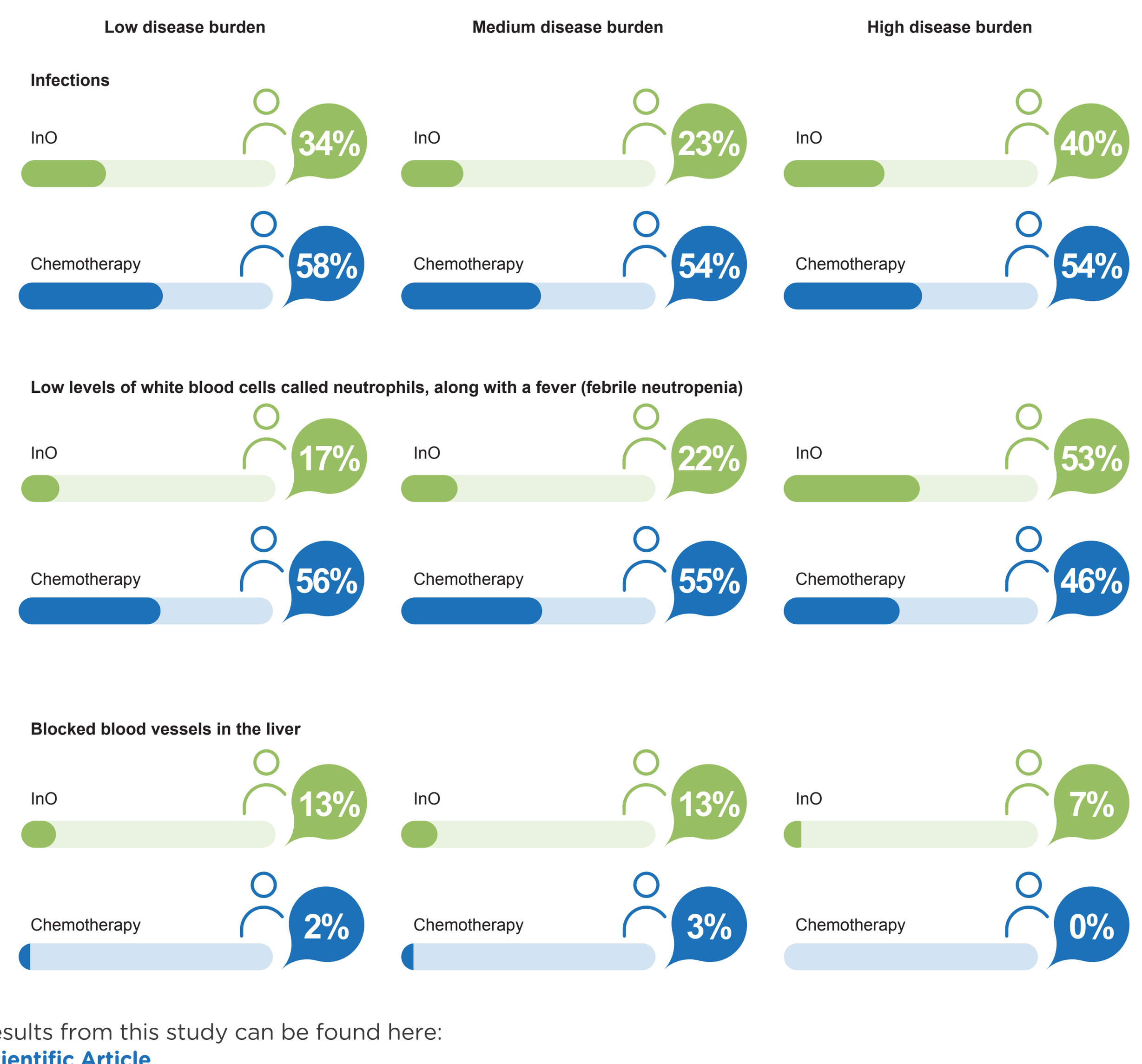

More results from this study can be found here:
View Scientific Article

What was the main conclusion reported by the researchers?

- In this study, compared with people who received standard chemotherapy, people who received InO were more likely to:
  - Achieve remission
  - Live to the end of the study
  - Reach the end of the study without their cancer getting worse.
- The researchers saw these results regardless of:
  - Whether people had a low, medium, or high disease burden
  - Whether they had extramedullary disease or not.
- For people who received InO, those with a high disease burden were equally as likely as those with a low disease burden to:
  - Achieve remission
  - Experience medical problems.
- For people who received standard chemotherapy, those with a high disease burden were less likely than those with a low disease burden to achieve remission.
- It is important to keep in mind that the number of people in the groups was small, making it more difficult to reach definite conclusions.

Are there any plans for further studies?

This study is completed.
There are studies currently underway looking at InO in:
- People who have not had any previous treatment for their ALL
- Combination with other treatments for ALL
- People with lymphoma
- Children and young adults with ALL.

Who sponsored this study?

Pfizer Inc, 235 East 42nd Street NY, NY 10017. Phone (United States): +1 212-733-2323.
Pfizer would like to thank all of the people who took part in this study.

Further information

For more information on this study, please visit:
View Scientific Article
https://clinicaltrials.gov/ct2/show/NCT01564784

For more information on clinical studies in general, please visit:
https://www.clinicaltrials.gov/ct2/about-studies/learn
http://www.cancerresearchuk.org/about-cancer/find-a-clinical-trial/what-clinical-trials-are

Plain language summary writing support was provided by Susan Tan, PhD, of Envision Pharma Group, Inc. and was funded by Pfizer.
