# Supplementary material for: Inotuzumab ozogamicin for relapsed/refractory acute lymphoblastic leukemia: outcomes by disease burden
Source: Blood Cancer J. 2020 Aug 7;10(8):81. doi: 10.1038/s41408-020-00345-8 (PMC7414105; doi:10.1038/s41408-020-00345-8)
Supplement: Supplementary file 2 — SI Methods and Tables S1-S4 [file 41408_2020_345_MOESM2_ESM.pdf]

Inotuzumab ozogamicin for relapsed/refractory acute lymphoblastic leukemia: outcomes by disease burden
Daniel J. DeAngelo,[1] Anjali S. Advani,[2] David I. Marks,[3] Matthias Stelljes,[4] Michaela Liedtke,[5] Wendy Stock,[6] Nicola Gökbuget,[7] Elias Jabbour,[8] Akil Merchant,[9] Tao Wang,[10] Erik Vandendries,[10] Alexander Neuhof,[11] Hagop Kantarjian,[8] and Susan O'Brien[12]

SI Methods Standard of care chemotherapy (SC) regimens

Patients in the SC arm received one of three regimens, at the investigator's discretion:

1. FLAG (fludarabine, cytarabine, and granulocyte colony-stimulating factor): In each 28-day cycle, patients received cytarabine 2.0 g/m^2 per day on days 1–6;
   fludarabine 30 mg/m^2 per day on days 2–6; and granulocyte-colony stimulating factor 5 μg/kg per day, in accordance with the standard of care at the institution. Patients were treated for a maximum of 4 cycles.
2. Cytarabine plus mitoxantrone: In each 15–20-day cycle, patients received cytarabine 200 mg/m^2 per day on days 1–7; and mitoxantrone 12 mg/m^2 per day on days 1–3. The mitoxantrone dose could be reduced to 8 mg/m^2 due to coexisting conditions, age, or prior use of anthracyclines. Patients were treated for a maximum of 4 cycles.
3. High-dose cytarabine: In one cycle of up to 12 doses, patients received cytarabine
   3 g/m^2 every 12 h. In patients ≥55 years of age, the dose could be reduced by up to 1.5 g/m^2. In patients >60 years of age, this dose was reduced to 1.5 g/m^2.

Modifications to these regimens were previously described[1].

Reference
1. Kantarjian HM, et al. Inotuzumab ozogamicin versus standard therapy for acute lymphoblastic leukemia. *N Engl J Med.* 2016; **375**: 740-753.

SI Table S1. Baseline patient and disease characteristics in the intent-to-treat population

	BMB <50%		BMB 50–90%		BMB >90%	
**Characteristic**	**InO (*n* = 53)**	**SC (*n* = 48)**	**InO (*n* = 79)**	**SC (*n* = 83)**	**InO (*n* = 30)**	**SC (*n* = 30)**
Age, median (range), y	49.0 (20–77)	52.5 (19–76)	46.0 (20–78)	45.0 (18–75)	38.0 (18–70)	46.0 (19–79)
Male, *n* (%)	23 (43.4)	25 (52.1)	48 (60.8)	53 (63.9)	18 (60.0)	23 (76.7)
Race, *n* (%)						
White	35 (66.0)	38 (79.2)	57 (72.2)	63 (75.9)	18 (60.0)	18 (60.0)
Black	1 (1.9)	1 (2.1)	3 (3.8)	1 (1.2)	0	1 (3.3)
Asian	12 (22.6)	5 (10.4)	10 (12.7)	14 (16.9)	9 (30.0)	5 (16.7)
Other	5 (9.4)	4 (8.3)	9 (11.4)	5 (6.0)	3 (10.0)	6 (20.0)
ECOG PS, *n* (%)						
0	23 (43.4)	21 (43.8)	28 (35.4)	30 (36.1)	11 (36.7)	10 (33.3)
1	26 (49.1)	18 (37.5)	44 (55.7)	45 (54.2)	9 (30.0)	16 (53.3)
2	4 (7.5)	9 (18.8)	7 (8.9)	8 (9.6)	10 (33.3)	3 (10.0)
Missing data	0	0	0	0	0	1 (3.3)
Salvage status, *n* (%)						
1	36 (67.9)	28 (58.3)	54 (68.4)	56 (67.5)	19 (63.3)	18 (60.0)
2	17 (32.1)	20 (41.7)	25 (31.6)	27 (32.5)	9 (30.0)	11 (36.7)
Missing	0	0	0	0	2 (6.7)	1 (3.3)
Duration of first remission <12 mo, *n* (%)	36 (67.9)	34 (70.8)	41 (51.9)	48 (57.8)	18 (60.0)	23 (76.7)
Response to last induction regimen, *n* (%)						
Complete response	40 (75.5)	31 (64.6)	60 (75.9)	64 (77.1)	20 (66.7)	16 (53.3)
Partial response	5 (9.4)	3 (6.3)	4 (5.1)	3 (3.6)	1 (3.3)	4 (13.3)
Stable disease	0	0	0	1 (1.2)	1 (3.3)	3 (10.0)
Resistant disease	8 (15.1)	12 (25.0)	13 (16.5)	12 (14.5)	7 (23.3)	6 (20.0)
Progressive disease	0	2 (4.2)	2 (2.5)	2 (2.4)	1 (3.3)	1 (3.3)
Unknown	0	0	0	1 (1.2)	0	0
Prior HSCT, *n* (%)	9 (17.0)	6 (12.5)	15 (19.0)	20 (24.1)	5 (16.7)	5 (16.7)
WBC, median (range), $10^3/mm^3$	3.5 (0.4–40.3)	4.7 (0.1–68.8)	5.2 (0.0–47.4)	4.4 (0.4–51.0)	3.2 (0.7–13.5)	2.4 (0.1–41.9)

Characteristic	BMB <50%		BMB 50–90%		BMB >90%	
	InO (*n* = 53)	SC (*n* = 48)	InO (*n* = 79)	SC (*n* = 83)	InO (*n* = 30)	SC (*n* = 30)
Peripheral blast count, median (range),[a] /μL	0 (0-27,779)	0 (0–43,331)	420 (0–42,660)	160 (0–31,500)	336 (0–11,700)	415 (0–31,425)
Peripheral blast count >0, *n* (%)	18 (34.0)	13 (27.1)	53 (67.1)	52 (62.7)	20 (66.7)	20 (66.7)
BMB, median (range), %	28.0 (5.0–48.4)	22.9 (5.0–47.0)	77.8 (50.0–90.0)	78.0 (50.0–90.0)	94.9 (91.0–100.0)	95.0 (91.0–100.0)
CD22 expression on ALL blasts, *n* (%)						
≥90%	35 (66.0)	25 (52.1)	54 (68.4)	47 (56.6)	17 (56.7)	21 (70.0)
≥70-<90%	8 (15.1)	5 (10.4)	15 (19.0)	12 (14.5)	6 (20.0)	0
<70%	2 (3.8)	6 (12.5)	2 (2.5)	9 (10.8)	1 (3.3)	3 (10.0)
Missing	8 (15.1)	12 (25.0)	8 (10.1)	15 (18.1)	6 (20.0)	6 (20.0)
Baseline cytogenetics, *n* (%)						
Normal	19 (35.8)	18 (37.5)	22 (27.8)	18 (21.7)	4 (13.3)	6 (20.0)
Metaphases analyzed ≥20	14 (26.4)	18 (37.5)	19 (24.1)	12 (14.5)	2 (6.7)	4 (13.3)
Ph+	9 (17.0)	8 (16.7)	10 (12.7)	14 (16.9)	3 (10.0)	4 (13.3)
t(4;11)	2 (3.8)	2 (4.2)	2 (2.5)	5 (6.0)	2 (6.7)	1 (3.3)
Complex	5 (9.4)	5 (10.4)	17 (21.5)	13 (15.7)	6 (20.0)	4 (13.3)
Del (9p)	0	2 (4.2)	1 (1.3)	1 (1.2)	1 (3.3)	0
Hyperdiploidy	1 (1.9)	1 (2.1)	5 (6.3)	1 (1.2)	1 (3.3)	0
Other abnormalities	7 (13.2)	4 (8.3)	13 (16.5)	16 (19.3)	9 (30.0)	9 (30.0)

[a] Values show peripheral blast count as measured on the first day of treatment, which may have been a few days after the day of randomization. Patients were excluded from the trial if they had peripheral blasts ≥10,000/μL on the day of randomization.

ALL: acute lymphoblastic leukemia; BMB: bone marrow blast; ECOG PS: Eastern Cooperative Oncology Group performance status; HSCT: hematopoietic stem cell transplantation; InO: inotuzumab ozogamicin; Ph+: Philadelphia chromosome-positive; SC: standard of care chemotherapy; WBC: white blood cell.

SI Table S2 Extramedullary disease cases at baseline

	Disease site	Lesion site
**InO arm**		
**1**	Lymph node	Submental adenopathy
**2**	Lymph node	Left supraclavicular, level 4; right cervical, level 3
**3**	Lymph node	Left subclavian region
**4**	Lymph node	Left supraclavicular, abdominal para-aortic, right inguinal, left inguinal, mesentery, axillary
**5**	Lung	Right lower lobe
**6**	Lymph node	Left axillary, right axillary, left axillary adenopathy, right axillary adenopathy
**7**	Kidney	Both kidneys
**SC arm**		
**1**	Posterior mediastinal	Posterior mediastinal
**2**	Ureteral mass	Retroperitoneal
**3**	Right paraspinal soft tissue mass	Paraspinal region
**4**	Breast	Right breast
**5**	Lymph node	Cervical, aortocaval, retroperitoneal

InO: inotuzumab ozogamicin; SC: standard of care chemotherapy

SI Table S3 Permanent and temporary discontinuations and dose reductions due to AEs in the safety population

	BMB <50%		BMB 50–90%		BMB >90%	
**AE, *n* (%)**	**InO (*n* = 53)**	**SC (*n* = 43)**	**InO (*n* = 79)**	**SC (*n* = 71)**	**InO (*n* = 30)**	**SC (*n* = 28)**
**Permanent discontinuations due to AEs[a], total**	12 (22.6)	2 (4.7)	13 (16.5)	5 (7.0)	6 (20.0)	4 (14.3)
Infections and infestations	4 (7.5)	2 (4.7)	4 (5.1)	1 (1.4)	2 (6.7)	3 (10.7)
Pneumonia	2 (3.8)	0	2 (2.5)	0	1 (3.3)	0
Sepsis	1 (1.9)	0	1 (1.3)	0	0	1 (3.6)
Hepatobiliary disorders	4 (7.5)	0	3 (3.8)	0	0	0
VOD/SOS	2 (3.8)	0	0	0	0	0
Hyperbilirubinemia	1 (1.9)	0	2 (2.5)	0	0	0
Investigations	2 (3.8)	0	1 (1.3)	0	2 (6.7)	0
AST increased	0	0	1 (1.3)	0	1 (3.3)	0
GGT increased	1 (1.9)	0	0	0	1 (3.3)	0
Blood and lymphatic system disorders	1 (1.9)	0	4 (5.1)	2 (2.8)	0	1 (3.6)
Febrile neutropenia	0	0	0	1 (1.4)	0	1 (3.6)
Thrombocytopenia	1 (1.9)	0	2 (2.5)	0	0	0
Neutropenia	0	0	1 (1.3)	1 (1.4)	0	0
**Temporary discontinuations due to AEs,[b] total**	27 (50.9)	5 (11.6)	34 (43.0)	8 (11.3)	10 (33.3)	4 (14.3)
Blood and lymphatic system disorders	18 (34.0)	3 (7.0)	23 (29.1)	1 (1.4)	4 (13.3)	0
Investigations	9 (17.0)	1 (2.3)	8 (10.1)	1 (1.4)	1 (3.3)	1 (3.6)
Infections and infestations	4 (7.5)	2 (4.7)	9 (11.4)	0	4 (13.3)	1 (3.6)
General disorders and administration site conditions	3 (5.7)	0	5 (6.3)	3 (4.2)	1 (3.3)	1 (3.6)
Hepatobiliary disorders	3 (5.7)	0	5 (6.3)	1 (1.4)	1 (3.3)	0
Musculoskeletal and connective tissue disorders	1 (1.9)	0	2 (2.5)	0	0	0
Psychiatric disorders	0	0	1 (1.3)	1 (1.4)	0	0
Gastrointestinal disorders	2 (3.8)	0	0	1 (1.4)	1 (3.3)	1 (3.6)
Vascular disorders	0	0	0	3 (4.2)	0	0
Cardiac disorders	2 (3.8)	0	0	0	0	0
Metabolism and nutrition disorders	3 (5.7)	0	0	0	0	0

AE, *n* (%)	BMB <50%		BMB 50–90%		BMB >90%	
	**InO (*n* = 53)**	**SC (*n* = 43)**	**InO (*n* = 79)**	**SC (*n* = 71)**	**InO (*n* = 30)**	**SC (*n* = 28)**
Respiratory, thoracic, and mediastinal disorders	2 (3.8)	1 (2.3)	0	0	0	0
Nervous system disorders	1 (1.9)	0	0	0	1 (3.3)	0
**Dose reductions due to AEs, total**	3 (5.7)	2 (4.7)	2 (2.5)	1 (1.4)	0	0

[a] AEs leading to permanent discontinuation of two or more patients are shown, along with the respective MedDRA system organ class.

[b] MedDRA system organ class shown if AEs within that class led to temporary discontinuation of two or more patients.

AE: adverse event; AST: aspartate aminotransferase; BMB: bone marrow blast; GGT: gamma-glutamyl transferase; InO: inotuzumab ozogamicin; MedDRA: Medical Dictionary for Regulatory Activities; SC: standard of care chemotherapy; VOD/SOS: veno-occlusive disease/sinusoidal obstruction syndrome.

Table S4. TEAEs of any grade occurring in ≥20% of patients in either treatment arm, in any BMB subgroup

	BMB <50%		BMB 50-90%		BMB >90%	
**AE, *n* (%)**	**InO (*n* = 53)**	**SC (*n* = 43)**	**InO (*n* = 79)**	**SC (*n* = 71)**	**InO (*n* = 30)**	**SC (*n* = 28)**
Any AE	52 (98.1)	43 (100.0)	79 (100.0)	71 (100.0)	30 (100.0)	28 (100.0)
Thrombocytopenia	29 (54.7)	30 (69.8)	41 (51.9)	42 (59.2)	11 (36.7)	15 (53.6)
Neutropenia	26 (49.1)	18 (41.9)	44 (55.7)	39 (54.9)	9 (30.0)	8 (28.6)
Anemia	13 (24.5)	27 (62.8)	30 (38.0)	37 (52.1)	11 (36.7)	14 (50.0)
Leukopenia	11 (20.8)	20 (46.5)	25 (31.6)	28 (39.4)	11 (36.7)	6 (21.4)
Lymphopenia	11 (20.8)	15 (34.9)	13 (16.5)	17 (23.9)	6 (20.0)	4 (14.3)
Febrile neutropenia	9 (17.0)	24 (55.8)	17 (21.5)	39 (54.9)	16 (53.3)	13 (46.4)
Pyrexia	17 (32.1)	18 (41.9)	24 (30.4)	29 (40.8)	10 (33.3)	12 (42.9)
Fatigue	14 (26.4)	4 (9.3)	19 (24.1)	18 (25.4)	7 (23.3)	2 (7.1)
Headache	12 (22.6)	9 (20.9)	24 (30.4)	24 (33.8)	9 (30.0)	5 (17.9)
Nausea	14 (26.4)	18 (41.9)	24 (30.4)	37 (52.1)	13 (43.3)	12 (42.9)
Diarrhea	9 (17.0)	17 (39.5)	17 (21.5)	27 (38.0)	4 (13.3)	11 (39.3)
Vomiting	5 (9.4)	10 (23.3)	14 (17.7)	19 (26.8)	6 (20.0)	6 (21.4)
Constipation	11 (20.8)	12 (27.9)	11 (13.9)	18 (25.4)	5 (16.7)	4 (14.3)
VOD/SOS[a]	9 (17.0)	1 (2.3)	11 (13.9)	2 (2.8)	3 (10.0)	0
Hyperbilirubinemia	10 (18.9)	8 (18.6)	17 (21.5)	11 (15.5)	8 (26.7)	5 (17.9)
GGT increased	19 (35.8)	5 (11.6)	11 (13.9)	5 (7.0)	5 (16.7)	2 (7.1)
AST increased	17 (32.1)	4 (9.3)	14 (17.7)	9 (12.7)	6 (20.0)	3 (10.7)
Hypokalemia	11 (20.8)	8 (18.6)	10 (12.7)	21 (29.6)	4 (13.3)	4 (14.3)

Data represent the safety population. AEs were graded according to the NCI CTCAE v3.0. Data shown are all-causality TEAEs with ≥20% incidence occurring in either arm (any treatment cycle, any BMB subgroup), and AEs of special interest.

[a] In July 2017 (after the clinical database was locked), a fourth case of VOD/SOS was confirmed in a patient treated with SC. This case of VOD/SOS occurred in March 2013, was not entered on the clinical report form, and is therefore not included.

AE: adverse event; AST: aspartate aminotransferase; BMB: bone marrow blast; GGT: gamma-glutamyl transferase; InO: inotuzumab ozogamicin; NCI CTCAE: National Cancer Institute Common Terminology Criteria for Adverse Events; SC: standard of care chemotherapy; TEAE: treatment-emergent adverse event; VOD/SOS: veno-occlusive disease/sinusoidal obstruction syndrome
