# Supplementary material for: Inotuzumab ozogamicin for relapsed/refractory acute lymphoblastic leukemia: outcomes by disease burden
Source: Blood Cancer J. 2020 Aug 7;10(8):81. doi: 10.1038/s41408-020-00345-8 (PMC7414105; doi:10.1038/s41408-020-00345-8)
Supplement: Supplementary file 3 — SI Fig. S2 [file 41408_2020_345_MOESM3_ESM.pdf]

Supplementary Fig. S2. Overall survival in responding patients in the InO arm, by bone marrow status and follow-up HSCT

A

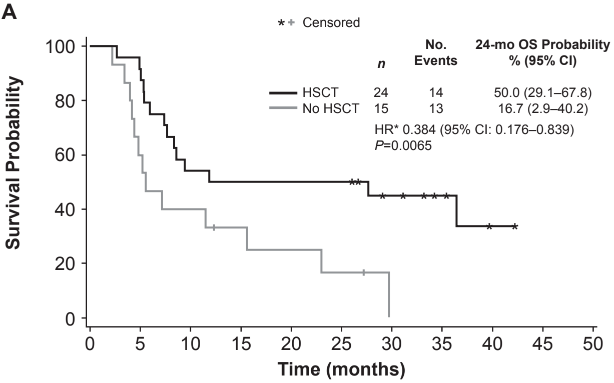

No. at risk											
HSCT	24	22	13	12	12	12	8	5	2	0	0
No HSCT	15	9	6	4	3	2	0	0	0	0	0

B

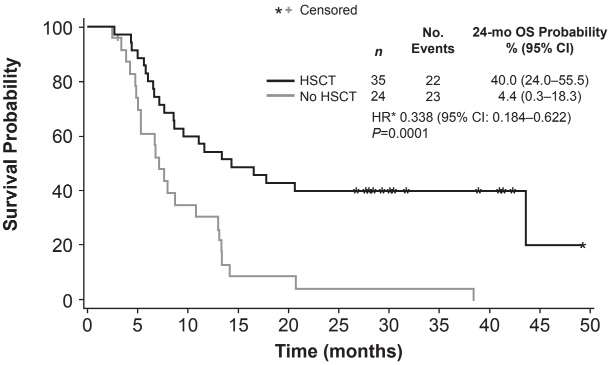

No. at risk											
HSCT	35	31	21	17	15	14	9	6	5	1	0
No HSCT	24	17	8	2	2	1	1	1	0	0	0

C

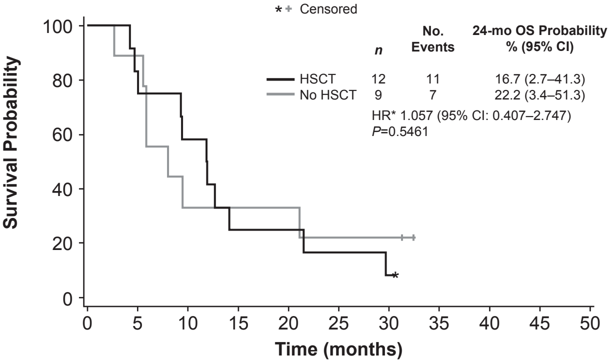

No. at risk											
HSCT	12	10	7	3	3	2	1	0	0	0	0
No HSCT	9	8	3	3	3	2	2	0	0	0	0

Kaplan-Meier plots show overall survival in the InO arm, among patients who achieved CR/CRi with BMB <50% (A), 50-90% (B), and >90% (C). InO, inotuzumab ozogamicin; HSCT, hematopoietic stem cell transplantation; OS, overall survival; HR, hazard ratio; CI, confidence interval; mo, months; CR, complete remission; CRi, CR with incomplete hematologic recovery; BMB, bone marrow blasts *HSCT vs No HSCT
